# Inflammatory Bowel Disease Nurse—Practical Messages

**DOI:** 10.3390/nursrep11020023

**Published:** 2021-04-01

**Authors:** Chiara Rosso, Alami Aroussi Aaron, Angelo Armandi, Gian Paolo Caviglia, Marta Vernero, Giorgio Maria Saracco, Marco Astegiano, Elisabetta Bugianesi, Davide Giuseppe Ribaldone

**Affiliations:** 1 Department of Medical Sciences, University of Turin, 10126 Turin, Italy; chiara.rosso@unito.it (C.R.); aaron.alamiarouss@edu.unito.it (A.A.A.); angelo.armandi@unito.it (A.A.); gianpaolo.caviglia@unito.it (G.P.C.); giorgiomaria.saracco@unito.it (G.M.S.); elisabetta.bugianesi@unito.it (E.B.); 2 Istituti Clinici Scientifici Maugeri, IRCCS, Gastroenterology Unit of Pavia Institute, University of Pavia, 27100 Pavia, Italy; mvernero@cittadellasalute.to.it; 3 Unit of Gastroenterology, Città della Salute e della Scienza di Torino-Molinette Hospital, 10126 Turin, Italy; mastegiano@cittadellasalute.to.it

**Keywords:** Crohn’s disease, communication, economy, education, fistulas, IBD, job, ostomy, pain, ulcerative colitis

## Abstract

*Background and Objectives*: Patients affected by inflammatory bowel diseases (IBDs) are complex patients with various problems from a clinical and psychological point of view. This complexity must be addressed by a multidisciplinary team, and an inflammatory bowel disease nurse can be the ideal professional figure to create a link between doctor and patient. The objective of this comprehensive review is to describe the figure of inflammatory bowel disease nurses and the various benefits that their introduction into a multidisciplinary team can bring, as well as a focus on how to become an inflammatory bowel disease nurse. *Materials and Methods*: A search on the PubMed database was performed by associating the terms “IBD” or “inflammatory bowel disease” with the Boolean term AND to the various issues addressed: “life impact”, “communication”, “fistulas”, “ostomy”, “diet”, “incontinence”, “sexuality”, “parenthood”, “fatigue”, “pain management”, and “follow up appointments”. Regarding the analysis of the benefits that the IBD nurse brings, the terms “IBD”, “inflammatory bowel diseases”, “Crohn’s disease”, and “ulcerative colitis” were used, associating them with the terms “benefit”, “costs”, “team”, and “patients”. Finally, regarding the focus on how to become an IBD nurse, an IBD nurse was interviewed. *Results*: An IBD nurse is a valuable nursing figure within the multidisciplinary team that takes care of patients with IBD because this nurse performs important functions from both a clinical assistance point of view (management of fistulas, ostomies, infusion of biological drugs) and an information and therapeutic education point of view (communication with patients, direct contact with patients by telephone or email). Furthermore, this nurse performs the “filter” function between doctor and patient, saving time for doctors that will be used for more outpatient visits. *Conclusions*: The introduction of an inflammatory bowel disease nurse is therefore recommended for multidisciplinary organizations dealing with the clinical course of patients suffering from IBD.

## 1. Introduction

Inflammatory bowel diseases (IBDs) are a group of diseases characterized by the presence of chronic inflammation in the absence of an infectious etiology. The main diseases included in this group are Crohn’s disease (CD) and ulcerative colitis (UC). These pathologies involve chronic inflammation of the intestine, characterized by phases of quiescence and phases of flare-up of the disease. The most common symptoms are abdominal pain, vomiting, diarrhea, flatulence, blood in the stool, frequent urge to evacuate with a sense of incomplete bowel emptying (tenesmus), and weight loss [1].

Since IBD is a systemic disease that can involve multiple organs or systems, patients with IBD often also have extraintestinal manifestations. In particular, joints, skin, and eyes are affected. The biliary tract, lungs, pancreas, and vascular system may be involved less frequently [2].

The patient with IBD, from a clinical point of view, is a complex patient, to be treated from a multidisciplinary point of view, namely, from a clinical point of view (diagnosing the disease, monitoring and treating signs and symptoms of the disease) and also from a psychological point of view. While many people with IBD are able to lead reasonably normal lives, an audit revealed that 88.5% of people with the disease feel that their quality of life is affected to some extent by the diagnosis and ongoing nature of the condition [3]. IBD often has an unpredictable course, with some patients experiencing rapid-onset intestinal and other symptoms exacerbations. These flare-ups can be very serious, require urgent care and sometimes hospitalization, and can even be life-threatening.

It has been suggested that important elements of care for IBD patients include quick access to clinics and providing adequate time to allow for discussion, explanation, information, and counseling; patients place a very high value on empathy, compassion, and interest [4]. Loss of energy, loss of control, low body image, isolation and fear, failure to reach full potential, and lack of information are the main concerns of people with IBD [5]. Patients with IBD who are parents of young children face many anxieties about their parenting, especially when they are unwell or hospitalized, and clearly need more practical help and support [6]. The patient wants to be seen as an individual, not as a diagnosis. They also want their experience with their condition to be recognized rather than ignored [7]. These psychosocial problems are often not the primary concerns of physicians, who, in their “medical model”, may focus more on diagnosis, disease treatment, and physical symptom management. It has been suggested that nurses are better suited to help and support patients with these problems [8] and that the team’s multidisciplinary approach should provide a more comprehensive care package. From these needs, the figure of the IBD nurse was born.

The objective of this narrative review is to research and deepen this nurse figure, who plays a very important role in filtering and communicating with patients and helps patients to manage their disease better.

## 2. Materials and Methods

A search on the PubMed database was performed by associating the terms “IBD” or “inflammatory bowel disease” with the Boolean term AND to the various issues addressed: “life impact”, “communication”, “fistulas”, “ostomy”, “diet”, “incontinence”, “sexuality”, “parenthood”, “fatigue”, “pain management”, and “follow up appointments”.

Regarding the in-depth analysis of the benefits that the IBD nurse brings, the terms “IBD”, “inflammatory bowel diseases”, “Crohn’s disease”, and “ulcerative colitis” were used, associating them with the terms “benefit”, “costs”, “team”, and “patients”.

Finally, regarding the focus on how to become an IBD nurse, an IBD nurse (E.S.) was interviewed.

## 3. IBD Nurse: Definition and Diffusion

The IBD nurse is a nursing figure who accompanies the patient with IBD in the organizational management of its care path. It has been advised by the British Society of Gastroenterology that the role of the IBD nurse practitioner should include several elements [9]:

- Connection between the patient and all members of the multidisciplinary team (and with other figures, if appropriate);
- Holistic support for the patient and their family in both the hospital and the community;
- Services managed by nurses: from a clinical point of view, telephone assistance, rapid triage, and organizational management of follow-up appointments;
- Support groups;
- Audit and research systems (e.g., patient database);
- Teaching and therapeutic education to patients, families, and other health professionals.

This nurse practitioner is now very widespread in northern European countries (mostly in the United Kingdom) and also in Canada and the USA [10]. This figure is prevalent in the most economically developed countries with very efficient healthcare: taking the European continent as an example, there is a greater diffusion of this nurse in Western Europe than in Eastern Europe. It is highlighted that specialist nurses play a very important role in reducing the psychological and emotional burden of IBD patients in these countries compared to Eastern European countries [11].

Today, in southern Europe, a nurse dedicated to IBD is an increasingly present reality in third-level centers. Where present, this nurse represents the reference point for every IBD patient under treatment, especially if in biological therapy.

The IBD nurse deals with various aspects of patient care, from communication and therapeutic education to the patient to the management of more complex problems such as fistulas or ostomies, passing through aspects of daily life that could influence the course disease, such as diet and sexuality.

## 4. Impact of Disease on Patient

In addition to symptoms such as diarrhea and fatigue, IBD commonly causes psychological distress to patients. The main concerns relate to the uncertain origins and course of the disease, execution of any surgery and/or creation of an ostomy, concern of not being able to reach the full potential of life, loss of bowel control, being a burden to others, production of unpleasant odors, and distortion of body image (due to, for example, the presence of an ostomy). Hospitalization can aggravate concerns about the potential for personal fulfillment by causing unwanted forced absence from work or studies.

Despite the fact that it is a major concern, IBD-related incontinence is rarely mentioned by patients to their doctor, but incontinence remains a major fear. According to new research, up to 74% of people with IBD experience fecal incontinence, which is not always linked to flare-ups [12]. During hospitalization, the problem of incontinence is not lessened, as a relapse is likely and the toilets can be shared among several patients, thus limiting their availability. The urgency can be serious, with some patients reporting a time delay of less than a minute between the urge in their bowels and the actual need to defecate. Loss of bowel function is so worrying that many IBD patients make a point of knowing where the closest bathroom is [13]. IBD nurses may provide empathic support and may be able to allow faster/easier access to facilities. In the event of an incontinence episode, discrete treatment and preservation of the patient’s integrity are critical. 

Health-related quality of life (HRQoL) is affected by IBD in both remission and relapse. While the effects have not been completely demonstrated, psychological intervention or therapy assistance could be useful for patients who are showing higher levels of concern [14]. Despite remission, underlying disease-related issues such as extraintestinal manifestations, fatigue, and sleep disorders may have an effect on HRQoL.

Patients affected by IBD also find that their illness has an effect on many facets of their lives, including relationships, education, socialization, and work. In a major European study conducted in partnership with patient organizations, more than 50% of patients with IBD indicated that their IBD had forced them to take time off work in the previous year, and almost 50% said that their IBD had stopped them from having a romantic relationship [15]. Furthermore, patients in this study often thought that their quality of life was not adequately discussed during visits as half reported that they could not talk about anything critical during a visit for their condition.

Many patients profit from interacting with those who are dealing with similar health issues. Sharing your story with people who “get” what it is like to live with a disease can be a source of vital social, mental, and psychological support [16]. Nurses may provide information about patient organizations and support groups. These are critical in offering specific, effective, and empathic support to people who are coping with a new diagnosis or complications from an existing disease.

## 5. Communication with Patient

Communication is an integral part of the nursing role, with both verbal and nonverbal skills being important in meeting the needs of the patient. IBD has a huge effect on patients’ lives and comes with a slew of unknowns. IBD nurses provide them with a great deal of encouragement, guidance, companionship, care, and empathy, which they value greatly.

Communication is critical in the formation of relationships and confidence with any chronic illness where the patient may have an ongoing interaction with healthcare professionals. It is critical to establish and sustain a therapeutic alliance between the nurse and the patient. It can be used to enable patients to self-manage, to take an active (rather than a passive) role in their own treatment, and to allow recognition while respecting the patient’s disease expertise [17].

Nurses should be aware that patients can struggle with the loss of their healthy self at different times. This can have an impact on how a newly diagnosed IBD patient offers, finds, receives, and processes information. Worry, anxiety, and their physical state will hinder their ability to interpret information, which must be conveyed in a way that is not easily misunderstood. To complement verbal information, written information, brochures, or web-based materials are recommended. Nurses may use patient information brochures produced by country-specific IBD patient organizations in the absence of in-house patient information brochures.

IBD nurses may be able to provide professional advice to patients about their IBD, but all nurses must be empathic and active listeners with enough expertise to offer practical guidance on key areas of concern to patients. Diet (with appropriate referral to specialized dieticians, who should be part of the IBD multidisciplinary team), social issues, common IBD symptoms and complications, IBD etiology, drugs and possible associated side effects, and surgical procedures are among these fields. IBD nurses may provide emotional support to patients by encouraging them to express their concerns. In a regular medical meeting, nonclinical conditions may be ignored, and patients will appreciate the opportunity to be heard and “taken seriously”. Nonspecialist nurses, on the other hand, are unable to offer guidance outside of their area of practice. It is possible to refer the patient to appropriate professional support such as IBD nurses, stoma care nurse specialists, dieticians, or counseling services if you are aware of them.

## 6. Management of Fistulas

The onset of fistulas is typical of CD, often occurring in the perianal region as communication between intestine and perianal skin or the abdominal wall or other organs.

Five important aspects for planning fistula management have been identified:

(1) identification or exclusion of local sepsis;
(2) evaluation of the nutritional status;
(3) anatomy;
(4) evaluation of the original intestinal loop;
(5) determination of the organs affected by the fistula and their contribution to the symptoms or to impairment of HRQoL.

Fistula control is still one of the most daunting activities for someone who deals with IBD patients. In order to treat these complications, a combination of medical, surgical, nursing, dietary, radiological, and other treatments may be needed. Enterocutaneous fistulas form as a result of a local inflammatory mechanism which is frequently worsened by surgery and may result in severe patient morbidity, such as electrolyte imbalances, abdominal pain, and sepsis. As a consequence, handling enterocutaneous fistulas is a daunting job for the multidisciplinary team (MDT), and it often necessitates referral to a specialist center.

The IBD nurse’s role in fistula management may include wound management, drug administration, sepsis containment, support, and bridging. The IBD nurse should not be expected to be an expert in complex wound management involving a medical and nonnursing approach. However, working collaboratively with appropriate healthcare professionals can help improve patient care and comfort.

Abscess drainage and seton insertion are standard surgical treatments for perianal fistulas, but in severe cases, diversion surgery or proctectomy may be necessary. One study looked at HRQoL in patients with serious perianal disease and discovered that, on average, HRQoL was higher in patients who had diversion surgery and/or proctectomy. However, it was also noted that the intervention could have an effect on such behaviors such as athletics, swimming, and sexual activity, emphasizing the importance of thorough debate and consideration that takes into account an individual’s unique circumstance [18]. Identifying the symptoms of a fistula and their effect on HRQoL will assist in the preparation of effective action. Additionally, identifying the underlying disease activity and resolving it, where possible, can also help improve outcomes.

Despite this, there are only a limited number of guidelines for nursing management of fistulizing disease, as fistulas are often refractory to both medical and surgical interventions and have a significant impact on HRQoL.

It is possible for an IBD nurse to recommend a redirection to patient support associations, the provision of information brochures, and, in specific situations, consideration of referral to more formal psychological counseling to help the patients manage their symptoms and impact on their daily life.

In short, in IBD-related fistulas, nurses play a role in ensuring patient comfort, protecting skin integrity, and managing complications. This can best be achieved by working in partnership with the broader MDT.

## 7. Ostomy Management

IBD patients who undergo ostomy surgery experience additional psychosocial barriers compared to patients who have IBD but who are not ostomy carriers. Several studies with small sample sizes suggest that these patients experience substantial postsurgical challenges such as decreased self-esteem, socialization, social stigma, sexual identity, independence, body image changes, embarrassment, pain, and loss of control. In addition, postsurgical stoma patients develop an immediate repulsion about their body and their stoma in the first few days after surgery upon seeing the bowel touch the skin [19].

The complications that a patient with an ostomy can face are many and are divided into complications of the ostomy itself and complications of the peristomal skin.

- Mucocutaneous separation

This can occur in the days following the formation of an ostomy. The stoma separates from the skin, leaving a visible space in the tissue. It can be caused by strain in the gut, infections, malnutrition, or the use of corticosteroids. It is important that fecal material from the stoma does not spill into the stoma’s surrounding region, as this poses a major risk of infection. The region must be properly medicated in order for the stoma to adhere to the skin and form a strong seal, preventing leakage. If the mucocutaneous separation is only superficial, it should be treated as required with a protective powder, seals, or paste. For deeper wounds, an alginate powder may be required.

- Retraction of the stoma

When the stoma is flush with the abdomen or retracts into the abdomen, the intestine should not protrude beyond the surface of the skin. This may be caused by a change in stoma size or shape, which may be linked to a patient’s weight gain or loss as well as intestinal stress or ischemia. Skin leakage and discomfort may occur as a result of retraction, as well as difficulty with adhering appliances to the skin. A convex lashing pouch can assist in the formation of a good seal and the prevention of leaks. The stoma must be closely controlled because of the extra pressure exerted on the abdomen by the appliance’s convex form as ulceration can occur.

- Bowel obstruction

Partially or fully obstructing ostomates are possible. Abdominal pressure, diarrheal leakage with a foul odor, abdominal distension, and stoma swelling are all symptoms of partial blockage. There will be no fecal material output from the stoma if it is completely blocked, which will be followed by extreme cramps, abdominal distention, nausea, and vomiting. Patients should be advised to avoid eating before the blockage has passed, maximize fluid intake, take a warm bath, and rub their abdomen to relieve pain in both cases. If the pain becomes intense or the patient begins to vomit, the patient should seek medical attention immediately.

- Stenosis

This happens as the stoma’s opening narrows. Ischemia, stoma retraction, CD, or radiation exposure to the intestine may all cause this. The IBD nurse will check the stoma and refer the patient to the surgical team, who will deepen the opening using a dilation procedure. If this does not solve the issue, surgery to reshape the stoma might be needed.

- Prolapse

A prolapsed stoma is one that spreads across itself, resulting in irregular lengthening. Weakened abdominal muscles, obesity, chronic constipation, or a chronic cough may all contribute to a prolapse. Surgery is typically not needed if the stoma is still working normally and has not changed color. A prolapse, on the other hand, can change the appearance of an ostomy, causing discomfort to the patient and making it difficult to use collection devices. Since a prolapse will grow in size over the day, it is critical that the collection bag’s opening can accommodate the changing size of the stoma. If a larger hole is required to accommodate the stoma, a barrier ring may be required to protect the peristomal tissue. Supportive garments should also be worn by the patient to help with prolapse and weakening muscles.

- Granulomas

These are red lumps that grow along the edge of an ostomy and can become irritated and bleed. They are not painful. Granulomas are caused by an ostomy pouch that does not fit correctly around the stoma and will continue to expand until the issue is resolved.

The most popular treatment is a barrier powder and seal/ring to protect the area; however, silver nitrate treatment may be necessary to minimize granulomas in some cases.

To keep the stoma in place, healthy peristomal skin is necessary. The key objectives of good stoma management are to avoid peristomal skin pain and to diagnose and treat any complications that arise early on as a variety of skin conditions may develop in the weeks and years after surgery. About 60% of patients with ostomates suffer from ostomy-related skin conditions [20].

- Contact dermatitis

This is the most common ostomy complication, and loss of liquid stools is reported as the cause in more than half of cases [21]. People with intestinal ostomies are at increased risk of developing skin problems due to corrosive enzymes that come into contact with the skin.

- Candida

It is characterized by very irritated, itchy skin and white pustules. For candida infections, topical antifungal creams should be used sparingly to ensure that the stoma pouch may still adhere to the peristomal tissue. If skincare products are interfering with pouch adhesion, a dermatological consultation may be needed.

- Peristomal gangrenous pyoderma

This is a very unpleasant skin disorder that accounts for just 0.5% of all peristomal skin conditions. It is characterized by ulcerative and pustular lesions/craters. Systemic steroid injections are used to treat it and are normally performed under the supervision of gastroenterological, surgical, and dermatological teams.

## 8. Diet Management

Patients also inquire about the relationship between eating habits and inflammatory disease symptoms. However, there is not enough conclusive epidemiological evidence to support diet as a risk factor. Patients with IBD should be aware of the importance of proper nutrition in preserving their health, particularly because they can lose weight during active illness. Dietary advice is usually best given by a dietician with a strong interest in IBD, who will also be part of the multidisciplinary team of an IBD center. It is critical that nurses, physicians, and surgeons (as well as patients) know this. Dietary guidance that is generic or uninformed can be frustrating for patients. However, certain general concepts are important for all members of the IBD team as well as patient awareness. 

There is no one-size-fits-all diet for all patients. It is uncertain if dietary changes help with symptom management alone or if they can help with full remission when used in conjunction with pharmacological agents. Patients should be encouraged to adopt a regular and balanced diet and lifestyle, as tolerated, since no special diet has been shown to be successful in treating IBD in adults. However, depending on the patient’s symptoms and preferences, it may be important to adjust their diet to suit their specific needs. For example, a patient with flare-ups of inflammatory disease may be advised to limit the use of legumes and foods that favor the formation of gas in the intestine, which can increase the sense of swelling and distension or limit the consumption of fiber and waste due to their ability to recall water and liquids inside the lumen of the intestine, thus increasing the already frequent diarrheal discharges. In the case of concomitant irritable bowel syndrome, stricture after surgery and ostomy formation, osteoporosis, anemia, or food allergies, detailed dietary guidance might be sought. 

For patients with IBD, having access to effective dietary assessments and expert advice is critical. Some patients may experience general malnutrition or specific nutritional deficiencies during their illness. Nutritional complications can be caused by a variety of factors, including drug–nutrient interactions, disease position, symptoms, and often insufficient dietary restrictions, all of which can have an impact on the patient’s health, nutritional status, and quality of life. This is not just true during active illness; long periods of recovery can reveal a wide variety of nutritional deficiencies [22]. Macronutrients (calories, proteins, and fats), vitamins (e.g., B12 and D), folic acid, and minerals (iron, calcium, magnesium, selenium, zinc) that are essential for anemia and osteoporosis are the most common nutritional deficiencies in IBD [23]. Complementary and alternative medicines, dietary supplements (vitamins, minerals, and trace elements), and herbal and homeopathic treatments should all be discussed with the treating physician.

Inadequate nutrition has the greatest effect on children and adolescents. Nutritional deficiencies can cause growth retardation, delayed puberty, bone demineralization, and serious psychosocial problems in children, so dietary considerations are especially important. Nutrition is an essential aspect of IBD management for infants. Nutritional therapy is the preferred treatment for active pediatric CD, in which a nutritionally full liquid diet meets all of the patient’s energy, protein, and other nutrient needs. Exclusive enteral feeding (EEN) is a treatment for small and large bowel disease that causes a response in more than half of cases. EEN is considered as a first-line therapy for children with active CD for a variety of reasons: it is a non-steroidal alternative to corticosteroid therapy that aids in the reversal of weight loss and growth failure, is better tolerated than steroids, and has a higher compliance rate than compared with EEN in adults [24]. Liquid nutrients, on the other hand, are often unsightly, so a small nasogastric tube should be used. The child and family will be taught how to insert and handle the tube as well as how to adhere to the feeding schedule. When a nasogastric tube is required for a school-aged child, correspondence should be made with the local community nurse and/or school nurse to ensure that therapy does not interfere with the child’s education.

## 9. Incontinence

Fecal incontinence (FI) can be a serious issue for IBD patients, affecting their physical, psychological, and social well-being. Patients with IBD who are afraid of incontinence may become housebound and unable to function. According to recent studies, FI is a serious yet underdiagnosed gastrointestinal condition in IBD. Patients with IBD report that FI is the most distressing symptom, but less than 50% of patients seek medical attention [25].

Patients often find it difficult to use the right words to openly reveal or discuss their intestinal symptoms. In order to do this by adequately addressing the problem of FI, the stigma that surrounds it must be overcome. Patients should be encouraged to seek help if they need assistance, and nurses can encourage patients to talk about continence problems simply by asking about symptoms. IBD nurses play a vital role in helping patients manage and improve symptoms of FI. One study showed that beginning infliximab therapy before anal sphincter repair surgery not only improved the severity of FI in patients with CD who had perianal complications but also preserved long-term continence in more than 50% of the subjects [26]. Nursing interventions in FI management may also include pelvic floor muscle exercises, perianal skincare, diet management, behavioral therapy (biofeedback), practical devices (including anal tampons), and electrical stimulation: two studies reported an improvement in FI in 100% of patients with sacral nerve stimulation (SNS) therapy [27,28]. The quality of life was also improved by SNS therapy. The IBD nurse must understand that not every patient can respond to the same nursing intervention. As a result, a customized care plan should represent the individual’s needs while still taking lifestyle factors into account.

## 10. Working Activity

The workplace is a very important concern for patients with IBD: the typical patient is a young patient who spends a lot of his time in the workplace. Disorders that IBD causes are very difficult to manage in a working context and can affect the productivity and self-esteem of patients, causing anxieties and fears about possible complications in the workplace (pain, incontinence).

A Canadian study [29] showed that more than a third of people with IBD report decreased work productivity in the workplace and presentism (a considerable part of the working time spent in personal activities, such as going very often to the bathroom), and strong associations were found between presentism and disability, lower quality of life, and emotional distress.

The rights that protect workers with IBD differ from state to state. The role of the IBD nurse is to inform the patient correctly and, in the absence of specialized information, refer the patient to employment agencies, trade unions, or even to specialized associations and IBD groups.

## 11. Sexuality

IBD most often affects people in their twenties and thirties, and it can have a serious impact on a person’s sexuality and self-confidence. HRQoL is determined by sexual function. IBD can affect a person’s body image, sexual function, and interpersonal relationships in both direct and indirect ways. Sexual issues can trigger anxiety and worry in patients with IBD.

Concerns over body appearance, urgency, or inappropriate losses during intercourse are examples of emotional aspects. Low self-image or self-esteem may result from the disease’s unpredictability and the fear of unexpected symptoms. Despite the lack of evidence in this field, the literature identifies high levels of sexual disability identified among both male and female IBD patients, with just over half indicating that their IBD had a negative effect on their relationship status [30]. Both males and females tend to be more affected by surgery, especially after a proctectomy [31]. Just over half of the respondents said their libido had diminished, and this was true for both CD and UC patients [32].

Nurses who identify problems with sexual function and sexuality must be able to support and report appropriate information. Intense workloads and staff changes can make it difficult to initiate discussions about sexuality in clinical practice; however, the nature of the nurse–IBD patient relationship can foster trust. Finding time to foster long-term relationships with patients is a significant aspect of the IBD nurse’s role.

The nurse’s role ranges from allowing sufficient time during visits to raise concerns, direct information, and offer advice to identifying when more structured support or specialist advice is needed. While there are no systematic tools for assessing the effect of IBD on a person’s sexuality, this will help to promote a more personalized approach to each circumstance.

Nurses must consider and feel comfortable addressing aspects of homosexual, lesbian, bisexual, and transgender (LGBT) patients’ sexual habits in order to help them overcome issues related to sexuality and chronic illness. Nurses who are under-informed about LGBT experiences in healthcare should consult the extensive literature on the topic.

## 12. Pregnancy

Fertility and pregnancy can be very emotional and sensitive times. Patients also express questions about topics such as possible heritability, substance use during pregnancy and breastfeeding, and the best mode of delivery when dealing with IBD. and fertility can be extremely emotional and sensitive times. Management of IBD can generate complexity, and patients often raise concerns about issues such as potential heritability, drug use during pregnancy and breastfeeding, or the best mode of delivery.

Research suggests a significant deficit in knowledge related to pregnancy in women with IBD [33]. The IBD nurse’s position within the team and the relationships they create with patients can often make them the healthcare provider with whom these issues are raised; they can provide a useful role in supporting and educating male and female patients in family planning stages, including contraception, during pregnancy, delivery, and after birth, along with alleviating concerns about other issues such as heritability, childbirth, or breastfeeding. In particular, for the latter, the latest recommendations [34] are clear: breastfeeding mothers with IBD should adhere to normal dietary guidelines. This includes raising their daily calorie intake and supplementing with omega-3 fatty acids from diet or supplements. However, keeping hydrated and well-fed can be difficult for mothers with IBD, particularly those who have an ostomy or who are losing weight due to active disease. In these situations, the mother should seek nutritional advice.

Women and men are frequently worried about the impact of disease-management drugs on fetal health. Nurses with the necessary expertise will assist patients in making informed decisions based on the risk–benefit profile of their specific case, in consultation with their doctor. Other services, such as the Food and Drug Administration (FDA), may be useful, but it should be noted that the FDA’s guidelines for the protection of certain drugs for pregnant IBD patients vary greatly from expert opinion on both sides of the Atlantic. Patients should discuss their pregnancy plans with their care team as early as possible so that interventions can be reviewed and optimized, if necessary, to improve maternal health. The IBD nurse will work with the patient during her pregnancy to reduce the risk of relapse and ensure that any therapy changes are made at the appropriate times. In more complicated patients, such as those with perianal disease, ostomies or those that are at high risk of potential colectomy, it is preferable that gastroenterologists and the obstetric team collaborate to make the best delivery decisions.

## 13. Therapeutic Education

Therapy for this type of patient is complex, and, according to a study, only 57% of patients with IBD have never skipped a single dose of mesalazine [35]. The factors that influence adherence to therapy are partly related to the prescribed therapy but also to factors that influence the patient’s life, so psychological support to the patient at the time of visit is also essential.

Self-management is a dynamic and interactive process that captures the complexity of living with a chronic disease in the context of everyday life [36]. Self-management includes skills that help people successfully manage chronic conditions on a daily basis [37]. Self-management education complements traditional patient education in supporting patients in order to live the best possible quality of life with their chronic condition. The role of the IBD nurse is crucial in improving a person’s self-confidence in their ability to handle their illness, which can help them cope with feelings of helplessness and humiliation.

## 14. Role of Nurses on Biological Therapy

It is probable that the role of the IBD nurse in biological therapy is currently the most widespread. The IBD nurse or, at least, a nurse dedicated to the administration of biological drugs intravenously over the years acquires great specific experience and is able to manage possible side effects (management of the infusion rate, allergic reactions) [38]. Furthermore, by being in contact for a prolonged time (for months or years) with the patient during infusions, the nurse forms a solid bond with the patient and becomes the first operator to whom the patient refers his problems. As for the administration of subcutaneous biological drugs, the IBD nurse instructs the patient, who will then become autonomous in home administration.

## 15. IBD Nurse and COVID-19

It is also important to measure the effect of the IBD nurse on disease outcomes and health care. Data from one study shows that in the first year after introducing an IBD nurse, the interventions performed resulted in the avoidance of 30 emergency room visits [39]. By scheduling an urgent clinical visit or exam within 1 week, the IBD nurse converted 30 emergency room visits to outpatient visits. During the study period, an additional 133 outpatient appointments were avoided through telephone or email counseling. Patients received “virtual care” by initiating or prescribing different amounts of drugs or referring the patient to their primary care physician for a physical exam with blood and/or stool sampling. These contacts were mostly assigned to the initiation, education, and follow-up of therapy, showing the impact of the IBD nurse in biologic monitoring and immunosuppressive therapy. Additionally, the IBD nurse provided faster access to IBD-related procedures and other departments for 136 patients. All of these data support the hypothesis that introducing an IBD nurse as part of the IBD multidisciplinary team improves a number of important quality indicators in managing IBD.

The presence of an IBD specialist nursing figure was found to be extremely helpful, especially during the COVID-19 pandemic that began in March 2020. In certain cases, planned follow-up appointments were canceled or rescheduled, and outpatient clinic access was restricted to patients who were psychotic or relapsing [40]. By the will of the patients or due to objective logistical difficulties, there has sometimes been a pause in the scheduled therapeutic infusions. Patients with IBD, particularly those on biological and immunosuppressive therapies, are thought to be at a higher risk of contracting SARS-CoV-2 [41]. For this reason, in a context of severely limited interactions between patients with IBD and therapists, many referral centers have begun to screen patients for COVID-19-related symptoms through telephone interviews or video calls, also managed in part by IBD nurses [42].

## 16. How to Become an IBD Nurse

In most of the world, the role of the IBD nurse is not yet officially recognized. Therefore, there is no real differentiation at the working level. However, the experience acquired in the management of these patients is recognized within the operating unit, and the nurse is involved in the therapeutic decisions to be made through participation in multidisciplinary meetings, in which the most difficult clinical cases are discussed. Various IBD centers have begun to understand the importance of having an IBD nurse in the center, so the number of people dealing with this pathology is increasing. For this reason, there has been an increase in opportunities to meet and conform through participation in training events in which opinions and experiences are also exchanged, which can help emerging centers to structure themselves.

At the moment, there is no training course for this figure, but IBD nurses learn “in the field”, thanks to the support of scientific societies, especially the ECOO, which has an entire section dedicated to the role of the IBD nurse. The ECCO has also published the “N-ECCO consensus statement”, which specifies the skills that an IBD nurse must acquire and also distinguishes the characteristics required for basic training and those required for advanced training [43]. On the other hand, as regards recognition at the contractual level, the role is currently not recognized.

## 17. Conclusions

In conclusion, the IBD nurse is a valuable nursing figure within the multidisciplinary team that takes care of patients with IBD because this nurse performs important functions from both a clinical assistance point of view (such as management of fistulas, ostomies, infusion of biological drugs) and an information and therapeutic education point of view (therefore, the part of communication with the patient, direct contact with the patient by telephone or email, and specific and personalized information suitable for each case), as well as an organizational function for the team and for the bureaucratic machine (management of the patient’s follow-up appointments, being a “filter” between doctor and patient, saving time for the doctor that will be used for more outpatient visits; Table 1).

According to the literature, the introduction of an IBD nurse is, therefore, recommended for multidisciplinary organizations dealing with the clinical course of patients suffering from IBD.

## Figures and Tables

**Table 1:** Roles of the IBD nurse.

Aspects of IBD	IBD Nurse Role
Impact of IBD on patient	Nurses caring for patients with IBD need to be aware of major patient concerns and the effect of IBD on quality of life.
Communication with patient	Communication is a two-way street. Nurses must learn to listen empathically and actively as well as provide important information about IBD and holistic care.
Fistula management	IBD nurses are responsible for patient comfort, skin integrity, and complication control. Working with a multidisciplinary team is the best way to address this.
Ostomy management	IBD nurse has a fundamental role in educating patients in ostomy care.
Diet management	Nurses need to be aware of potential nutritional problems in patients with IBD to ensure they are properly identified and managed, especially in specific situations such as after surgery.
Incontinence	Nurses should evaluate the impact of incontinence on health-related quality of life.
Working activity	IBD nurse has the role of an information agent regarding the management of IBD-related symptoms and the presence of rights of the worker with IBD, as well as referral to specialized associations or patronages.
Sexuality	Sexuality-related issues can cause anxiety and concern in IBD patients. IBD nurses must be able to advise and report on patients who have issues with sexual function and sexuality.
Pregnancy	Before conception, during pregnancy, and after birth, IBD nurses must provide guidance and information to patients and partners. It is likely that patients will need to talk with other medical professionals. If at all practicable, discussions about pregnancy should begin before conception.
Therapeutic education	IBD nurses must instruct patients and their caregivers based on individual needs, preferences, and coping skills. The goal is to empower patients to live with the disease.

IBD, inflammatory bowel disease.
